# Five Ways in Which Computational Modeling Can Help Advance Cognitive Science: Lessons From Artificial Grammar Learning

**DOI:** 10.1111/tops.12474

**Published:** 2019-10-30

**Authors:** Willem Zuidema, Robert M. French, Raquel G. Alhama, Kevin Ellis, Timothy J. O'Donnell, Tim Sainburg, Timothy Q. Gentner

**Affiliations:** ^1^ Institute for Logic, Language and Computation University of Amsterdam; ^2^ LEAD‐CNRS, University of Burgundy; ^3^ Max Planck Institute for Psycholinguistics; ^4^ Department of Brain and Cognitive Sciences MIT; ^5^ Department of Linguistics McGill University; ^6^ Department of Psychology University of California San Diego; ^7^ Department of Psychology & Division of Biological Sciences University of California San Diego

**Keywords:** Computational modeling, Neural networks, Formal grammars, Bayesian modeling, Artificial language learning, Artificial grammar learning

## Abstract

There is a rich tradition of building computational models in cognitive science, but modeling, theoretical, and experimental research are not as tightly integrated as they could be. In this paper, we show that computational techniques—even simple ones that are straightforward to use—can greatly facilitate designing, implementing, and analyzing experiments, and generally help lift research to a new level. We focus on the domain of artificial grammar learning, and we give five concrete examples in this domain for (a) formalizing and clarifying theories, (b) generating stimuli, (c) visualization, (d) model selection, and (e) exploring the hypothesis space.

## Abstract 2

Zuidema et al. illustrate how empirical AGL studies can benefit from computational models and techniques. Computational models can help clarifying theories, and thus in delineating research questions, but also in facilitating experimental design, stimulus generation, and data analysis. The authors show, with a series of examples, how computational modeling can be integrated with empirical AGL approaches, and how model selection techniques can indicate the most likely model to explain experimental outcomes.

## 1 Introduction

Computer models have given us a better understanding of everything from the evolution of stars to the evolution of the human eye, from chemical reactions in the ozone layer to animal mating behavior, and much more. Over the years many computational models have been developed for the study of cognition. The first computer model (Rochester et al., 1956) of category learning had of a total of 69 “artificial neurons” and cranked out its calculations at the rate of 5,000 computations/second (compared to 93 × 10^15^ computations/second for the fastest computer today). Many models have been developed since, contributing to a better understanding of some of the processes underlying human cognition. If nothing else, they revealed human cognition to be far more difficult to simulate computationally than had been previously suspected.

Despite this long history in cognitive science, computational modeling is not uncontroversial. Computational modeling sometimes appears as an inward looking field—a domain separated from experimental research, where obscure technical details dominate, hungry for data but seldom giving something back. Modeling, in that view, is a post hoc process, taking place after data collection and, at best, providing an implementation of explanatory theories of experimental results.

However, this is not how things need to be. Computational techniques, even simple ones that are straightforward to use, can greatly facilitate designing, implementing, and analyzing experiments, and generally help lift research to a new level. In this paper, we give five concrete examples of how computational models can help design and implement experiments, as well as help in analyzing and interpreting the results. We focus on the domain of Artificial Grammar Learning (AGL), a field that employs artificial language stimuli to systematically manipulate certain factors to test for language learning. AGL is a particularly illustrative case because the design of artificial stimuli capturing particular features of natural language, while ruling out other interpretations, is particularly challenging. It is also a field where many of the same types of debates have happened as in cognitive science at large, and where many of the different computational modeling paradigms have been applied (e.g., Alhama & Zuidema, 2017, 2019; Culbertson et al., 2013; Frank et al., 2010; French et al., 2011; Gagliardi et al., 2017; Kemp et al., 2007; Kirby et al., 2015; Marcus et al., 1999; Pearl et al., 2010; Perruchet et al., 2006; Wonnacott, 2011).

In the remainder of this paper, we will discuss our examples in an order that roughly follows the experimental cycle. We start where, ideally, all research starts, with rival theories on the cognitive processes underlying grammar learning. In Section 2, we discuss how models can be used to formalize and clarify theories. In Section 3, we shift to implementations of concrete experiments. As computational tools for generating stimuli, presenting stimuli, and recording responses are well‐known, we focus on the use of computational techniques for selecting and randomizing stimuli and for avoiding confounds in the experimental design. When the data are collected, the next task is to analyze and report the results. In Section 4, we discuss modern computational techniques, such as those coming out of the deep learning field, that go beyond such standard reporting and visualization techniques, and offer great insight into the cognitive systems under study. In Section 5, we discuss the use of model selection techniques for exploratory data analysis that allows one to uncover patterns in the data not easily discernible without computational modeling techniques. Finally, in Section 6, we discuss how a system based on Bayesian Program Learning can be used to explore a space of hypotheses, by generating and visualizing alternative hypotheses on strategies that participants in an AGL experiment might follow.

## 2 Formalization

One of the most important uses of computational techniques is to define formal, explanatory models. In this section, we will illustrate the benefits of having a formal and computationally implemented model available, using the “word segmentation” phenomenon, from artificial language learning, as our running example. We know that children as young as 2 months of age can segment “words” from a continuous syllable or image stream devoid of any markers indicating word boundaries and that they can do this without recourse to semantics. How?

There are two main, and conflicting, views of how infants do this. The first says that they have a mechanism that is sensitive to the probabilities of hearing one syllable and expecting it to be followed by another (i.e., Transitional Probabilities). Boundaries between words are where these probabilities are lowest. Another view says that infants remember hearing certain pairs of syllables (“chunks”) better than other pairs because they occurred more frequently in the syllable stream. They automatically build internal representations of these frequently heard pairs and incorporate these internal representations to build larger syllable *chunks*.

Both views make intuitive sense and rely on a body of empirical work. Formal modeling can help in evaluating which of the views provide a better explanation for the empirical record as a whole, by first making both views more precise, by evaluating whether they qualitatively reproduce the data equally well, and by deriving new testable predictions.

The Transitional Probability view has been formalized by the well‐known Simple Recurrent Network (SRN) of Elman (1990). Formalizing the alternate Implicit Chunk Recognition view has yielded a model called TRACX2 (initially introduced by French & Cottrell, 2014, and based on an earlier version of the same architecture: TRACX; French et al., 2011). Both models can be used to fulfill the task of segmenting sequences of sounds, images, movements, and so on into “words,” in a bottom‐up manner. And both the SRN and TRACX2, it turns out, can successfully *reproduce existing data* when tested on syllable streams such as those used in early infant artificial language learning experiments (e.g., see Mareschal & French, 2017).

**Figure 1:**
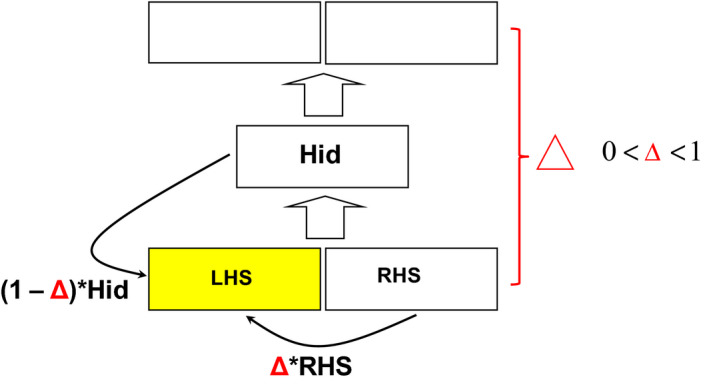
Architecture and information flow in TRACX2. Let *S* be the sequence of phonemes, syllables, images, or movements be designated by *S* = {*S*_1_, *S*_2_, …, S*_t_*, *S_t_*_+1_, …, *S_n_*}, where each *S_i_* is a vector representing the *i*th phoneme, syllable, and so on in the sequence. At time *t* + 1, the right‐hand bank (RHS) of input units is filled with the next input: RHS*_t_*_+1_ = *S_t_*_+1_ The left‐hand bank (LHS) of input units is filled with a blend of the right‐hand input and the hidden unit activations at the previous time step: LHS*_t_*_+1_ = (1 − $\Delta_{t}$) × Hid*_t_* + $\Delta_{t}$) × RHS*_t_*. $\Delta_{t}$ is the hyperbolic tangent (tanh) of the absolute value of the maximum error across all output nodes at time *t*, Hid*_t_* are the hidden unit activations at time *t* and RHS*_t_* is the activation across the right‐hand bank of input nodes at time *t*.

We will not discuss details of SRNs or TRACX2 here (Fig. 1 gives a succinct description of TRACX2). One important aspect of both models is that they receive information item‐by‐item and try to integrate the new information with information from previous items in a compressed representation: the hidden layer. SRNs are typically trained to predict the next item based on their output; TRACX2, in contrast, attempts to reproduce the current input based on its output.

TRACX2 is thus a *recurrent*, *autoencoder* network (“recurrent” means that it processes sequences item‐by‐item, and information from previous time steps stays in the network through recurrent connections; “Autoencoder” means that it is trained to produce on output what appears on input).

Crucially, if TRACX2 is successful (low reproduction error), much of the compressed representation will be maintained for the next time step. If the reproduction error is high, most of the compressed representation is discarded. Key to understanding the model's behavior is the observation that if at time *t*, the reproduction error ($\Delta_{t}$) is small, this could only have occurred if the network has seen the two items together on input many times (otherwise $\Delta_{t}$ could not be small).^1^ In lay terms, this means that as you experience short subsequences of items (auditory, visual, tactile) over and over again, these items become bound to each other more and more strongly into a chunk until we no longer perceive its component parts.

In the process of building a formal model, we are forced to become much more *precise* about the principles that we believe underlie the phenomenon of interest. And formal, explanatory models offer more benefits. Crucially, the availability of a computational implementation allows us to derive *new*, *testable predictions*. TRACX2 makes testable predictions about what should occur when the number of words in the syllable stream increases, when the length of words increases, when word frequencies in the input stream follow a Zipfian distribution, when syllable data are replaced by visual data, and so on. All of these predictions can, and have been, tested (Frank et al., 2010; Kurumada, Meylan, & Frank, 2013; Mareschal & French, 2017).

One major difference in predictions derived from SRNs and TRACX2 is worth considering in some more detail: the predictions about backward transitional probabilities. To illustrate what is meant by backward TPs, consider the domain of letters (rather than syllables) and words, specifically the letter pair “ez” in French, as in “Parlez‐vous français?” The letter “e” is followed by a “z” only 3% of the time (forward TP = 0.03). However, “z” is preceded by an “e” 84% of the time (backward TP = 0.84). Both adults (Perruchet & Desaulty, 2008) and infants (Pelucchi et al., 2009) can use backward transitional probabilities to segment syllable streams. This leads to a prediction—namely, that SRN models, which implement forward TPs, should fail on these data, but TRACX, based on its memory of chunks of syllables seen on input, should have no problem with backward TPs. This is precisely what happens when the models are tested on these data.

Another key property of implemented, computational models is that we can modify the parameters at will, potentially discovering unexpected new behaviors that, indirectly, lead to new predictions. We refer to this as *probing the model*. The TRACX2 architecture has a number of parameters that can be probed. One of the key parameters that was modified in TRACX2 was the rate at which it learns. By varying the learning rate (i.e., the amount that the synapses are modified on each presentation of new data on input), Mareschal and French (2017) were able to closely model the evolution of chunking in 2‐month‐old, 5‐month‐old, and 8‐month‐old infants (Slone & Johnson, 2015).

In conclusion, a key use of computational techniques is to build formal, explanatory models for a real‐world phenomenon, *P*. Most useful models satisfy at least five fundamental criteria—namely, (a) they are based on principles thought to undergird *P*, (b) they are able to qualitatively reproduce data generated by *P*, (c) they provide a human‐understandable explanation of *P*, (d) they make testable predictions about new data generated by *P*, and (e) they can be “probed,” by which means that the parameters of the model can be modified and the results of those modifications can be used to make further predictions (Cleeremans & French, 1996). Such models help bring research to a new level, by suggesting new directions for empirical research and by helping us choose between alternative theoretical positions.

The precision that comes with formalizing models also makes them sometimes vulnerable to criticism, as not all design choices can always be supported with independent evidence; even this vulnerability is, however, a strength rather than a weakness, as the choices are at least made explicit. The process of formalization is already helpful in our understanding of the real differences between alternative accounts.

## 3 Generating stimuli

The key component of AGL experiments is the use of artificial language stimuli, for which we choose its basic units and the rules to combine them. Generating stimuli that contain only the regularities of interest is, usually, not at all trivial. In this section, we discuss how computational techniques can help (a) avoid implicit biases, (b) prevent confounds, and (c) allow for more complex studies.

Let us begin with (a). When manually selecting the stimuli, there is no principled way to ensure that the implicit knowledge of the researchers is not biasing the stimuli selection. Researchers possess very specialized knowledge, in addition to their awareness of the goals of the experiment. In some cases, they can even predict the responses to each stimulus: Forster (2000) showed that researchers can accurately predict lexical decision reaction times of participants after screening test items. Applying automatic randomization procedures is required to remove the bias.

As for (b), it is clearly difficult to generate stimuli that only have systematic variability on the dimension under study, and unfortunately, confounds are often discovered after the experiment has already been done. For instance, one of the seminal papers in AGL (Marcus et al. 1999), aimed to uncover the acquisition of grammar‐like structures in infants, but the initial experiment was found to contain another systematic variation at the level of phonetic features that could have guided the results, and thus a second experiment had to be reported. Similarly, Peña et al. (2002) investigated the learnability of nonadjacent dependencies between syllables, but a phonological pattern (Onnis et al., 2005; Seidenberg et al., 2002) as well as the insertion of silent pauses (Perruchet et al., 2006) was shown to influence the results.

Computational models do not fully guarantee that there will be no confounds in the stimuli—after all, the type of patterns we want to rule out need to be prespecified—but they do capture the usual suspects. As an example, Beckers et al. (2016) present four measures for characterizing auditory stimuli based on certain forms of overlap between training and test stimuli that are deemed to be highly salient.

Finally, point (c): using computational techniques can lift our experiments to the next level, since hand‐crafting the stimuli unnecessarily constrains the complexity of the experiment. To illustrate this, we briefly discuss an AGL experiment that could not have been carried out without the help of computational techniques. Elazar et al. (unpublished data) aimed to study whether the statistical relations between syllables in participants' native language (L1) influence their segmentation of an unknown (artificial) language. The experiment involved two conditions: one in which participants were familiarized with an artificial language made of words that were statistically *consistent* with L1, and another in which they were *inconsistent*. Consistency was defined in terms of the frequency of stimulus bigrams in L1.

The authors computed several statistics from a corpus of L1: the frequencies of syllable bigrams within words, transitional probabilities, and relative frequencies of onset syllables. These statistics were then used to select candidate stimuli. Given a candidate triplet of the form ABC (where A, B, and C are consonant–vowel syllables), the summed frequency of the bigrams AB and BC should be higher than a threshold τ_1_ for consistent words (and lower than τ_2_ for inconsistent words), but AB, BC, and ABC should not be actual words in L1. For instance, “nibemo” might be a consistent word, since both “nibe” and “bemo” are frequent bigrams in the L1 of the study (Spanish), but they are not words in that language and neither is “nibemo.”

Both consistent and inconsistent words are made of the same syllables, and the syllables occupy the same position in the triplet (A, B, or C), but for each consistency class, only triplets which do not have overlapping syllable bigrams are selected. This means that, to find eight words of each consistency class, the number of triplet candidates amounts to 8^3^ = 512 for one class and 8 × 7 × 6 = 336 for the other. From all these triplet candidates, eight words need to be selected for each consistency class, so that each syllable appears only once in each set. Therefore, before applying the frequency constraints for each consistency class, there are $\left(\begin{array}{c} 336\\ 8 \end{array}\right)$ candidate sets, a number that goes over three quadrillion.

Solving this problem thus entails navigating a huge space of possibilities, from which only a few meet all the constraints. Fortunately, computer scientists have developed algorithms for these type of problems, known as *constraint satisfaction problems*. The use of a simple algorithm of this kind (*backtracking*) makes it possible to search this vast space of possibilities in order to find a set of words that satisfy all of the constraints. This computational technique was also applied to the generation of the foils in the experiment, which had similar characteristics. In this way, the authors managed to select stimuli required for a study that could not have been carried out without the use of such computational techniques (both for the computation of the frequencies in L1 and for the final selection of the stimuli).

## 4 Synthesis and visualization

Well‐designed AGL studies can provide powerful tools to investigate explicit cognitive capacities related to processing different grammatical patterns or rules, and when applied in comparative model organisms, they have the potential to reveal explicit neurobiological mechanisms. However, often overlooked is the domain specificity of these cognitive capacities, and how they may (or may not) interact with the elements that constitute AGL sequences. Many studies investigate whether humans or nonhuman animals can learn “ABB,” “XYX,” or “AnBn” patterns, but the way in which our X's, Y's, A's, and B's are instantiated—as auditory or visual signals, as tones or speech‐like stimuli, as vocalizations of their own or of another species, as alarm calls or as social signals—might be crucial depending on the cognitive mechanisms involved.

Indeed, patterns in the real world often differ from the sequences used in AGL studies in that the former comprise high‐dimensional and temporally continuous elements that are poorly described by discrete, well‐defined categorical representations. As AGL studies mature, it is incumbent on the field to better understand the relationship between real‐world categories and grammatical (or other) sequencing rules in order to make AGL tasks less artificial, but this is not always easy. For comparative AGL studies with birdsong, for example, creating naturalistic acoustic sequences typically requires experienced humans able to identify hundreds of unique categories of song syllables or motifs, and different people rarely agree on all the segmentation and categorization decisions.

To address these challenges, computational models can be of great help, in particular techniques for dimensionality reduction and generative modeling. *Dimensionality reduction* refers to the ability to project very high‐dimensional signals into a low‐dimensional space, while preserving the local structure and similarity of the original high‐dimensional representations. The underlying assumption of dimensionality reduction is that the original high‐dimensional space is sparsely filled, and that most of its structure can be retained by projecting local relationships onto a lower dimensional space. When prior knowledge exists about how to reduce dimensionality (as in the case of human speech), it should be used, of course. For many natural signals, however, the information allowing dimensionality reduction is not available. Fortunately, many modern algorithms, such as Principal Component Analysis (PCA), t‐Distributed Stochastic Neighbor Embedding (t‐SNE), and the convolutional autoencoder, that we will discuss here, do not require prior knowledge of relevant dimensions and can be used to project high‐dimensional stimuli in a low‐dimensional space based upon the structure of the full signal distribution.

*Generative modeling* refers to a style of models that can generate new data, and probability distributions over possible outcomes (which in turn can be used to define the likelihood of the model producing data identical to empirically observed data). In our quest to design behavioral and physiological experiments that exploit the rich feature spaces of natural signals, we are greatly aided by such generative models, especially if there are parameters we can manipulate that regulate the likelihood of the observed data. Autoencoders (such as also introduced in Fig. 1) are an attractive tool for this purpose because they also produce a generative model of the input data. In other words, while classical dimensionality reduction techniques map data from the original high‐dimensional space (*X*) to a low‐dimensional space (*Z*), generative models such as autoencoders can also do this the other way around (from *Z* to *X*) and generate new stimuli that closely resemble the original stimuli. Due to this property, autoencoders can be used to simultaneously gain insight into the distributional properties of complex natural signals in a low‐dimensional space, and to generate systematically (smoothly) varying stimuli in the original high‐dimensional signal space.

**Figure 2:**
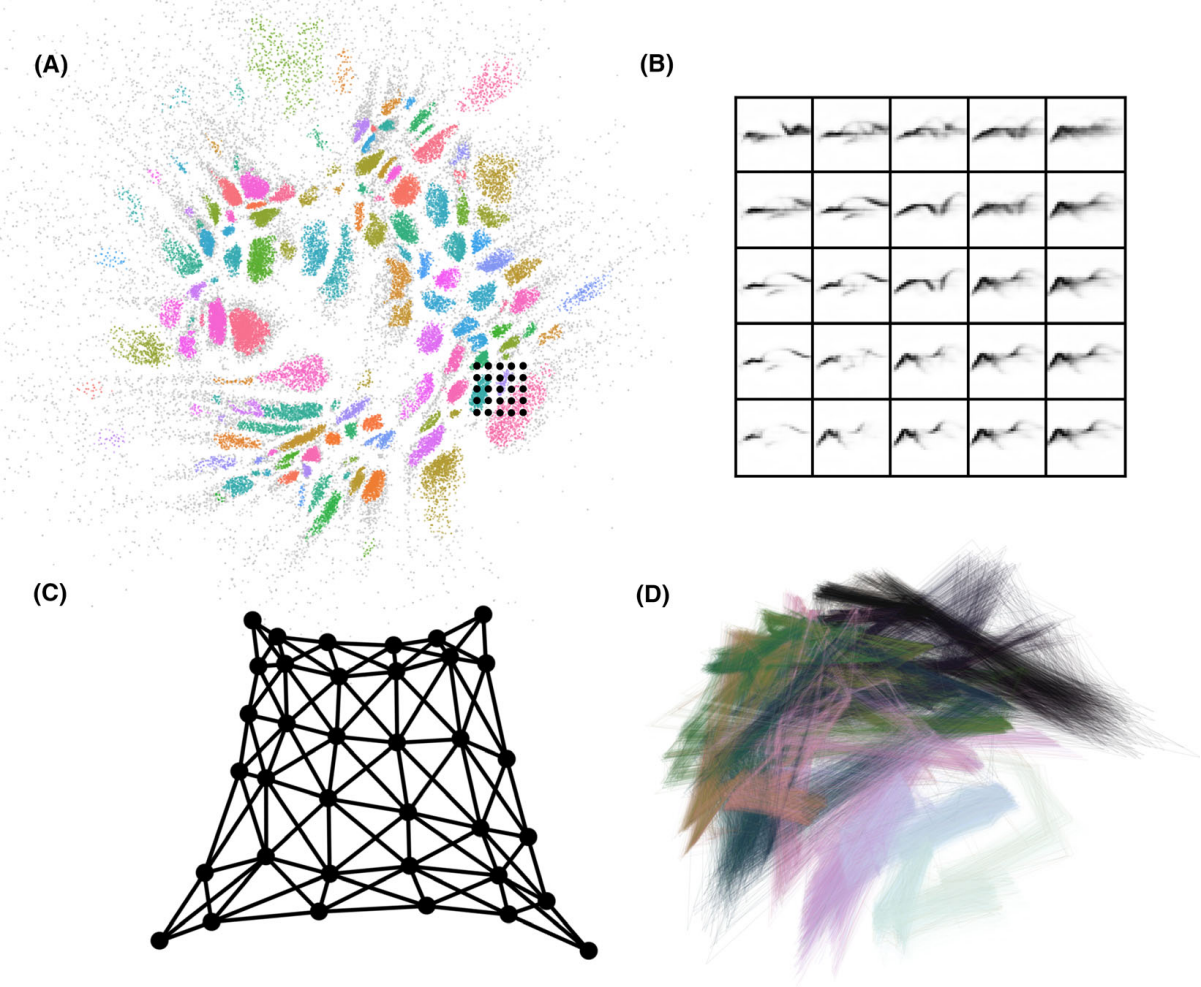
Neural network projections of birdsong vocalizations into a 2D latent space. (A) A scatter plot where each point in 2D space represents a syllable sung by a Cassin's vireo (library acquired from Hedley, 2016). Colors denote hand labeled syllable categories, which tend to cluster in the low‐dimensional space. The 5 × 5 grid in the lower right quadrant marks the locations of samples drawn from the 2D space. (B) Spectrograms of synthetically generated syllables, corresponding points in the 5 × 5 grid in (A), where each spectrogram is produced by projecting the 2D points into the decoder network. (C) A similar uniform grid, sampled from a 2D plane of a different neural network trained on European starling song. Signals generated from each point in the grid are presented to a different starling trained on a same‐different operant conditioning task. Distances between points on the grid, and thus the overall warping, reflect an empirically measured similarity between neighboring syllables. (D) A plot of transitions between syllable clusters in a 2D space similar to (A) but from a single European starling; transitions between sequential elements are shown as lines. Line color shows the relative time of a syllable transition within a bout; later transitions are darker.

As an example, Sainburg et al. (unpublished data) trained a convolutional autoencoder on a large sample of 1,024‐dimensional spectrographic representation (32 × 32, frequency × time bins) of syllables from a birdsong corpus. Each original syllable is projected onto a 2D space (Fig. 2A) where each colored dot represents a single syllable from a bird's song. Conversely, every point in this low‐dimensional space corresponds to a “song‐like” syllable, whether produced by the bird or not. Sampling systematically from the 2D space (black grid in Fig. 2A) and projecting each point back through the network, produces systematically varying stimuli in the original high‐dimensional input space (Fig. 2B). High‐dimensional stimuli generated from the network in this, or other systematic ways, can be used for behavioral or physiological playback experiments. For example, in one behavioral task, we computed the perceptual similarity between generated syllables from a 2D grid in a low‐dimensional manifold and used a same–different two‐alternative choice task to map perceptual similarity onto that grid (Fig. 2C).

Combining dimensionality reduction with density‐based unsupervised classification techniques can reveal unbiased decision boundaries between putative signal categories that follow the distributional statistics of the high‐dimensional inputs. An example of this is shown in Fig. 2A, where each point is color coded according to the output of an unbiased density‐based classifier (McInnes & Healy, 2017) that has discretized the songs of several birds (in this case Cassin's vireos). Large libraries of natural vocal signals discretized in this way can be used to directly measure and compare longer timescale sequential properties (Fig. 2D), such as transition statistics, across large corpora from diverse taxa.

In sum, dimensionality reduction is a powerful tool for discovering structure in distributions in an unbiased manner. Similarly, generative modeling can provide an unbiased method for quantitatively controlled sampling from natural signal distributions. When combined, these two techniques provide a powerful framework for visualizing and producing sequences of naturally varying, but categorically well‐defined, signals that can be patterned by grammatical or other rules. These techniques decrease reliance upon an experimenter's a priori knowledge and assumptions, replacing qualitative perceptual intuition with a quantifiable stimulus space, rendering AGL studies more realistic and ultimately more powerful.

## 5 Model selection

When an artificial grammar experiment is performed and all the data are gathered, we would like to know which of the (often many) plausible hypotheses best explain the data.

Before analyzing the data, however, we distinguish between two types of data analysis: confirmatory and exploratory. In a confirmatory data analysis, ideally we follow a preregistered protocol to minimize the degrees of freedom of the analysis and maximize its statistical power. These protocols specify how we cluster the data and measure the statistical significance of observed differences between conditions or the goodness of fit of alternative hypotheses. Techniques for doing this are, of course, part of the standard toolbox of experimental scientists (although preregistration and Bayesian data analysis are still less popular than they perhaps should be).

In exploratory data analysis, by contrast, we have much more freedom, as long as we indicate clearly that we are in an exploratory phase. It is in this phase that computational modeling can be most useful, in particular, through model selection. If we specify our hypotheses in terms of concrete computational models, we can compute how well each of them fits the complete pattern of data: not just the main dependent variables, such as “fraction of positive responses” or “percent correct” in a block of trials, but also the kind of errors made, the reaction times, and the evolution of responses over time.

A simple example is the exploratory data analysis in Van Heijningen et al. (2009) which studied the ability of zebra finches to detect a “context‐free” pattern in an artificial grammar learning experiment. Birds were trained in a Go–NoGo paradigm to respond to stimuli with an *A^n^B^n^* pattern and reject stimuli with a (*AB*)*^n^* pattern (or vice versa). Data were gathered to establish whether the birds had, indeed, acquired an *A^n^B^n^* “rule,” but the results proved inconclusive at the population level. Then to explore the data further, the authors defined a number of simple computational models that implemented alternative hypotheses on what individual birds could have acquired. These models included the hypothesis that birds had acquired a rule to look for the BA transition and, if detected, reject the stimulus (NoGo). For each stimulus, the model computes the *likelihood* of performing a “NoGo” as:

(1) $$\begin{array}{c} P\left(\text{Action}=\text{NoGo}|\text{Acquired}\;\text{Rule}=\text{not}\;-\;\text{BA}\right)\\ =\left\{\begin{array}{cc} \left(1-\epsilon \right) & \text{if}\;\text{stimulus}\;\text{contains}\;\text{BA}\\ \epsilon & \text{otherwise} \end{array}\right. \end{array}$$

where ϵ is a “noise” parameter that specifies how likely it is that a bird performs a “Go” despite having internalized a rule that prescribes “NoGo.” The likelihood of the entire data sequence of one bird during the test phase under the given hypothesis is simply the product of likelihoods of each stimulus–response pair. Van Heijningen et al. computed for each bird the likelihoods under each of a range of different hypotheses and concluded that for none of the birds the *A^n^B^n^* hypothesis was the maximum likelihood hypothesis. Although the models in Van Heijningen et al. were very simple (they could be implemented with a single line of code), they allowed the authors to highlight a pattern in the data that remained hidden in the population‐level analyses. Their approach to highlighting individual differences between birds was subsequently applied successfully to bird learning abilities in the visual domain (Ravignani et al., 2015).

A more complex example of the use of a model selection approach in artificial language learning can be found in a series of papers by Frank et al. (2010), French et al. (2011), and Alhama and Zuidema (2017). These papers evaluated three very different and rather complex models on the same set of data from human subjects, collected in an online experiment by Frank et al. (2010). Frank et al. propose to look at how performance of human participants (measured with a forced choice task) improves or degrades with three manipulations: varying sentence length, varying number of tokens, and varying vocabulary size. They find that their favored model, the Bayesian Lexical Model (with a “forgetting” option), does give a better *correlation* to the human data than a number of baselines they define. French and Cottrell (2014) and Alhama and Zuidema (2017), in turn claimed that their own models gave even higher correlations. The outcome of this debate is not yet settled. For the current paper it suffices to observe that the combination of (a) a formalization of alternative theories as computational models, (b) a common dataset, and (c) a common model selection criterion allows us to perform detailed, *quantitative* comparisons of different theoretical explanations for the collected data.

Where van Heijningen et al. used *likelihood* and Frank et al. used *correlation* as the sole criterion, many other criteria for evaluating goodness of fit are proposed in the literature. Many of these alternatives behave somewhat similarly to likelihood, or use likelihood as one component (and add an extra component to favor simpler models over more complex ones, or favor models that are otherwise a priori already more probable). Which criterion to use depends on why model selection is being done in the first place (exploration, confirmation), the nature of the hypotheses set one is considering (e.g., for choosing between 10 discrete hypotheses one needs different methods than for selecting optimal parameters on a continuous scale), as well as personal taste (see Claeskens, 2016 for a good overview). Ultimately, the results of these analyses are most convincing if multiple criteria point in the same direction, and most revealing if the criteria allow us to distinguish between competing models and highlight qualitative differences between them (Alhama et al., 2015).

## 6 Exploring the hypothesis space

In the previous section we considered “Bayesian model selection” as a way for the scientist to choose between multiple hypotheses about observable data. In this section we generalize this notion and show that we can also use Bayesian concepts to explore an entire space of hypotheses. We consider the case of an AGL learner exposed to different word forms, for example, those in Gerken (2010) and Marcus et al. (1999),  and discovering rules or regularities in that input.

First, we need to formalize this situation, and we do this using formal grammars to describe the rules, and Bayesian tools to describe the learning problem. The *grammars* are context‐sensitive rewrite rules (Chomsky & Halle, 1968), as commonly used in generative phonology. Each rewrite rule is a function that both inputs and outputs a sequence of phonemes. These rewrites are written as “input → output/left_right,” which means that “input” gets rewritten to “output” whenever “left” is to the left and “right” is to the right. Rules can refer to sets of phonemes by writing down vectors of phonological features; rules can also bind variables to phonemes or syllables.

In the Bayesian learning setup, we (a) place a prior distribution over grammars, for example, a distribution that puts more probability mass on shorter or simpler grammars; (b) equip each grammar with a *likelihood model* that specifies exactly how likely a grammar is to produce a given utterance; and then (c) use Bayes's rule to work backwards from the utterances to the grammar that was likely to produce them. A simple and intuitive prior over grammars is one which penalizes longer grammars and favors parsimonious grammars, for example, defining *P*(grammar) ∝ exp(−# symbols in grammar). An example of a likelihood model, and the one we use here, is just to count the number of extra bits or symbols needed to encode an utterance given the grammar. We can think of Bayes rule as a recipe for scoring how well a grammar explains AGL stimuli. In practice, the space of all grammars is infinite and combinatorial, so we also need an efficient procedure for searching the space of grammars.

Using this framing and the mathematical tools from Program Synthesis (e.g., Ellis et al., 2015, unpublished data), we can study how the human mind deals with the trade‐off between grammars that are *a priori* probable (the prior prefers small grammars; “parsimony”) and grammars that assign high likelihood to the utterances (and therefore closely “fit the data”). For example, a learner could infer a grammar that just memorized the utterances (perfect fit but poor parsimony) or it could infer a grammar that can generate every possible word (parsimonious but a poor fit).

Even for the simple word forms used in AGL experiments, there is a massive space of possible grammars that learners could explore. In classic model selection, we use our computational tools to produce a single best grammar—a single hypothesis on the grammar a child might acquire in such an experiment. But what that optimal grammar is will depend on how the child solves the trade‐off between parsimony and fit to data, and we often do not know exactly how those competing criteria are weighted. The formalization above, however, allows for an even more interesting analysis: We can explore the entire space of possible grammars and visualize all those grammars that are optimal solutions to the trade‐off given some weight; that is, we search for the set of grammars that are not worse than another grammar along the two competing axes.

**Figure 3:**
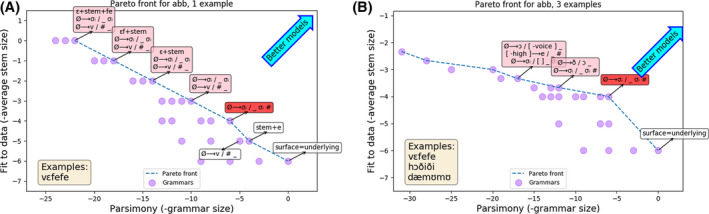
Pareto fronts for the ABB (Marcus et al., 1999) learning problem for either one example word (left) or three example words (right). Rightward on *x*‐axis corresponds to more parsimonious grammars and upward on *y*‐axis corresponds to grammars that best fit the data, so the best grammars live in the upper right corners of the graphs. Red shade: ground truth grammar. Pink shade: shares structure with ground truth grammar. White shade: incorrect generalizations. As the number of examples increases, the Pareto fronts develop a sharp kink around the ground truth grammar, which indicates a stronger preference for the correct grammar.

This set is called the **Pareto front** (Mattson & Messac, 2005). Intuitively, grammars on the pareto front are ones which an ideal Bayesian learner prefers, *independent* of how the learner decides to relatively weight the prior and likelihood. Fig. 3 visualizes the Pareto fronts for two AGL experiments as the number of example words provided to the learner is varied. What these Pareto fronts show is (a) the set of grammars entertained by the learner, and (b) how the learner weighs these grammars against each other as measured by the prior (parsimony) and the likelihood (fit to the data).

As an example of how the Pareto front visualizes the space of possible generalizations, consider the left Pareto front in Fig. 3 (*aab, 1 example*). Here the learner has seen a single word, [vεfefe]. Some grammars living on the Pareto frontier are as follows:

- **A grammar that generates every possible word:** In the lower right‐hand corner of Fig. 3 is the grammar labeled “surface=underlying,” which just says that every word (a “surface pronunciation”) is represented (“underlyingly”) literally how it is pronounced. The observed word [vεfefe] is represented as /vεfefe/, which has six symbols, giving a fit to the data of −6.
- **A grammar that duplicates syllables:** Highlighted in dark red in Fig. 3 is a grammar with the single rule $\emptyset \to \sigma_{i}/\_\sigma_{i}\#$ which inserts a copy of the last syllable. This rule generates the word [vεfefe] by starting with the stem (i.e., underlying form) /vεfe/ (which has four symbols, fit to data of −4) and then applying the rule in the grammar, which copies the last syllable and makes [vεfefe].
- **A grammar that duplicates syllables and appends morphemes:** Highlighted in pink in Fig. 3 are grammars that duplicate a syllable but also append or prepend extra morphemes. These correspond to generalizations where the learner believes that different parts of [vεfefe] correspond to prefixes or suffixes in the language. For example, the grammar in the upper left corner incorporates the suffix /fe/ as well as the prefix /vε/, and explains the observation using an underlying form that is completely empty (fit to data of 0)—this grammar has memorized the observed data, and so maximally compresses it, but at the cost of having many symbols inside of the grammar (22 symbols, vs. 6 symbols in the grammar that just duplicates a syllable).

The shape of a Pareto front suggests the most likely generalization an AGL participant would make given the stimuli, which can be used to select a set of stimuli. Contrasting the Pareto fronts to the right and left in Fig. 3, one sees a sharp kink around the target generalization as the stimuli more strongly single out the intended generalization (right plot: stimulus set contains more data). So, by visualizing the space of plausible generalizations for each stimulus set, we can select those that would push an ideal Bayesian learner to make the generalizations under consideration.

Grammar induction is an underconstrained problem, and in general there are infinitely many grammars that could explain a collection of utterances. Despite this ambiguity, both children and linguists can make plausible inferences about which grammars best explain a collection of utterances, not only in phonology but also in other aspects of grammar, such as semantics (Piantadosi, 2011), syntax (Perfors et al., 2011), and morphology (O'Donnell, 2015). The computational tools shown here offer a generic way of formalizing, exploring, and visualizing the range of alternative hypotheses, and explaining why a child, linguist, or computer program might prefer one over the other.

## 7 Conclusions

We have argued in this paper that computational techniques can take cognitive research, in general, and artificial grammar research, in particular, to a new level. We have attempted to highlight the value of integrating computational modeling with empirical approaches to AGL. Modeling is not a separate field to be done in parallel and independent of experimental research. Modeling is also not a post hoc activity that takes results from experimental work and gives them a new twist. Rather, we have attempted to show that computational techniques and empirical research can, and should, go hand in hand, each reinforcing and strengthening the other.

In particular, we have discussed how computational modeling techniques can be used for *clarifying theories*: the process of formalization forces us to specify details of our theories that would otherwise have remained vague, and the formalized (and implemented) models allow us to potentially derive unexpected consequences from our assumptions—as discussed in Section 2, using the example of the phenomenon of “word segmentation” in artificial language learning experiments.

In addition, we discussed the role models play in suggesting new experiments: We can use computational models to derive new, testable predictions (Sections 2 and 4), to generate stimuli for experiments (Sections 3 and 4), and even to generate new, testable hypotheses (Section 6).

Finally, we pointed to the useful function of models to give us novel insights about experimental data: by providing visualization techniques that show structure not visible with standard techniques (Section 4), and by allowing us to test the goodness of fit of a range of alternative models to the data (Section 5).

## Note

1 In this case, the contribution of the hidden unit activations, which constitute the network's internal representation of the two items on input at time *t*, to RHS*_t_*_+1_ will be large, because tanh($\Delta_{t}$) will be close to zero; conversely, if $\Delta_{t}$ is large, meaning that the items on input have not been seen together often, the hidden layer's contribution to LHS at time *t* + 1 will be relatively small because tanh($\Delta_{t}$) will be close to 1, meaning that 1 − tanh($\Delta_{t}$) will be close to zero).
